# Cardiometabolic Care: Assessing Patients with Diabetes Mellitus with No Overt Cardiovascular Disease in the Light of Heart Failure Development Risk

**DOI:** 10.3390/nu15061384

**Published:** 2023-03-13

**Authors:** Christina Chrysohoou, Christos Fragoulis, Ioannis Leontsinis, Ioannis Gastouniotis, Dimitra Fragouli, Maximos Georgopoulos, Emmanouil Mantzouranis, Marina Noutsou, Konstantinos P. Tsioufis

**Affiliations:** 1 1st Cardiology Clinic, Hippokration Hospital, National and Kapodistrian University of Athens, 11528 Attica, Greece; 2 Diabetes Center, 2nd Department of Internal Medicine, Medical School, National and Kapodistrian University of Athens, Hippokration General Hospital of Athens, 11528 Athens, Greece

**Keywords:** diabetes mellitus, heart failure, biomarkers, risk

## Abstract

The mechanisms leading to the development of heart failure (HF) in diabetes mellitus (DM) patients are multifactorial. Assessing the risk of HF development in patients with DM is valuable not only for the identification of a high-risk subgroup, but also equally important for defining low-risk subpopulations. Nowadays, DM and HF have been recognized as sharing similar metabolic pathways. Moreover, the clinical manifestation of HF can be independent of LVEF classification. Consequently, approaching HF should be through structural, hemodynamic and functional evaluation. Thus, both imaging parameters and biomarkers are important tools for the recognition of diabetic patients at risk of HF manifestation and HF phenotypes, and arrhythmogenic risk, and eventually for prognosis, aiming to improve patients’ outcomes utilizing drugs and non-pharmaceutical cardioprotective tools such as diet modification.

## 1. Introduction

Diabetes mellitus (DM) is a global health problem. The prevalence of DM worldwide continues to increase, with a projected rise from 425 million in 2017 to 629 million by 2045 globally. The effects of this pathological entity on cardiovascular health (CV) create further public health challenges and remain responsible for the increased prevalence of cardiovascular disease (CVD) [1]. Beyond coronary artery disease (CAD) progression, DM can precipitate or worsen heart failure (HF) due to multifactorial mechanisms (i.e., the accumulation of advanced glycation end products (AGEs), oxidative stress, inflammatory status impairment, decay of intracellular calcium, changes in microRNAs expression) [2,3,4]. Patients with diabetes develop HF at more than two-times the rate of patients without diabetes [1]. The underlying mechanisms that interconnect DM to HF merit further investigation, although diabetic cardiomyopathy and the frequent concomitant presence of microvascular dysfunction have been implicated as the principal insults. Nowadays, DM and HF have been recognized as sharing similar metabolic pathways; thus, the clinical manifestation of heart failure can be noticed in the entire spectrum of ejection fraction [5]. Thus, HF approach should be through structural, hemodynamic and functional evaluation, while the need for the early detection of functional and morphological alterations in the cardiovascular system in diabetic patients has emerged. Therefore, the necessity comes forth for evidence-based cardioprotective tools, apart from drugs, such as the implementation of a Mediterranean diet [6]. 

## 2. Major Interconnections between DM and HF

Nowadays the concept of HF has been disconnected from the presence of reduced ejection fraction [7], while symptoms and signs of HF can be acknowledged in the whole spectrum of left ventricular (LV) volume exchange, which is prescribed as LV ejection fraction (LVEF). Dichotomizing function using LVEF is a major oversimplification, as patients who present with a small LV cavity size and ventricular hypertrophy, or significantly impaired longitudinal contraction may also develop low flow status. HF can be present with normal or reduced end-diastolic volume or even with increased end-diastolic volume but reduced stroke volume [8].

### 2.1. Structural Cardiac Alterations

DM has been recognized as sharing similar pathophysiological pathways with HF, even in the absence of clinical recognition. In clinical diabetic cardiomyopathy, we usually recognize two faces: the dilated and the restrictive one. The first one may be due to extended coronary atherosclerotic disease or due to the inflammatory process predisposing to dilatation [9,10], while the second one may be due to microvascular dysfunction, infiltration, fibrotic accumulation or even low metabolic state [11]. 

### 2.2. Energetic Impairment

DM causes several alterations in cardiac energy handling and myocardial performance [12]. In the presence of any disturbance of glucose metabolism, cardiac adenosine triphosphate (ATP) production is affected. Under normal circumstances, the major energy-providing substrates for the heart are fats (triglycerides and fatty acids) and carbohydrates (glycogen, glucose and lactate). Depending on its environment, the heart selects the most efficient fuel for respiration, and continues likewise even when stressed (adaptation). The absence or the overabundance of any fuel may result in metabolic toxicity and contractile dysfunction [13]. The failing heart relies on an exogenous substrate for energy provision due to the lack of endogenous energy reserves. It has been presented that even if the heart is unable to synthesize creatinine, its contractile performance can be preserved under rest conditions. However, this performance is reduced when challenged with an inotropic agent; this can be attributed to increased susceptibility to ischemic injury and electrical instability, which in turn can also lead to a state of low metabolism, where the intrinsic myocardial performance is depressed [14]. 

### 2.3. Other Contributors of HF Development in DM

DM rarely presents in isolation. Not infrequently, diabetic patients concomitantly present with several other cardiovascular risk factors such as obesity or arterial hypertension (HTN) [15].

Obesity affects myocardial structure and performance through systemic inflammation, epicardial adipose tissue (EAT) accumulation and consequently the development of the clinical condition described as “Cardiac steatosis”. Furthermore, myocardial fibrosis, augmented production of reactive oxygen species (ROS) and reduced production of nitric oxide (NO) by endothelium and increased myocyte stiffness are several mechanisms behind cardiac dysfunction in diabetes [12]. In obese type 2 diabetes mellitus (T2DM) patients, the unfavorable metabolic environment characterized by hyperglycemia, lipotoxicity, abundance of AGEs and hyperinsulinemia can induce coronary microvascular dysfunction, and lead to the development of heart failure with preserved ejection fraction (HFpEF) [8]. Hyperglycemia causes impairment in endothelial NO generation and a reduction in the production of cyclic guanosine monophosphate (cGMP), which in turn down-regulates protein kinase G (PKG) activity in cardiomyocytes and consequently titin protein function, causing diastolic distensibility. Similarly, AGEs impair endothelial NO production and predisposes to concentric LV remodeling and myocardial stiffness as observed in diabetic cardiomyopathy patients with HFpEF [8,12]. In addition, in T2DM there is an increase in glucose autooxidation and free fatty acid concentrations, which can create oxidative stress in the myocardium and consequently induce concentric ventricular remodeling [3].

The EAT exhibits a direct interplay with the heart, in metabolic and mechanica aspects. The lack of muscle fascia between the EAT and the myocardium makes the two tissues dependent on the same microvasculature, whereas, on the other hand, it allows direct paracrine and vasocrine interactions. In fact, it has been shown that in obese patients, the EAT secretes several proinflammatory chemokines and cytokines, collectively called adipokines (including among others, tumor necrosis factor alpha (TNF-α), monocyte chemoattractant protein-1 (MCP-1), interleukin (IL)-6, IL-1β, plasminogen activator inhibitor-1 (PAI-1), resistin and S100 calcium-binding protein A9 (S100A9). All together, they create a proinflammatory state in the myocardium associated with cardiomyocyte stiffness, coronary endothelial dysfunction and fibrosis, which are all implicated in the development of HFpEF [16].

Moreover, high levels of ROS produced by the EAT are responsible for the oxidative stress in the myocardium and the coronary vasculature. As a source of angiotensin II, the EAT triggers coronary vasoconstriction leading to ischemia, especially in patients with DM due to coexisting vasculopathies [17]. Metabolically, EAT expansion is associated with intramyocardial accumulation of triglycerides causing cardiac steatosis. It seems that myocardial triglyceride content is independently associated with reduced pumping function and impaired ventricular strain parameters. Cardiac steatosis induces fetal gene transcription that favors the utilization of myocardial glucose instead of free fatty acids, which further aggravates lipid accumulation, even under physiological conditions [16,17,18].

## 3. Diabetic Cardiomyopathy as a Standalone Disease

Diabetic cardiomyopathy can represent an important solitary etiology of HFpEF in patients without coronary artery disease, arterial hypertension or other forms of structural heart disease [11]. Despite not being fully elucidated yet, it seems to share similar molecular pathways of impaired energy utilization leading to low performance status.

Those patients diagnosed may have the dilated or restrictive phenotype with concentric LV remodeling and diastolic LV dysfunction and even low levels of natriuretic peptides (NP). Patients with HFpEF and normal NP levels usually display mild diastolic dysfunction and preserved cardiac output reserve during exercise, despite the marked elevation in filling pressures. While clinical outcomes are not as poor compared with patients with high NP, patients with normal NP HFpEF exhibit increased risk of death or HF readmissions compared with patients without HF. These patients also reveal worse right ventricular function, which is associated with low levels of NP, and, more often, secondary valvular regurgitation. The increased morbidity and mortality that they show in comparison with patients without heart failure, emphasize the importance of this phenotype [19,20,21].

## 4. Arrhythmogenic Considerations in DM

The pathophysiology of ventricular arrhythmias in non-ischemic cardiomyopathy (non-ICM) involves complex remodeling of both ventricles with interstitial and perivascular patchy fibrosis (extensive endocardial scarring is detected by magnetic resonance imaging (MRI)) in 33% of DCM patients, with a propensity for epicardial and mid-myocardial layers, intertwined with surviving myocytes, localized necrosis and cellular infiltrates [22]. In addition to the altered electrophysiological properties of hibernating/stunned myocytes around the scar, fibroblasts themselves have been found to affect ionic currents by providing mechanoelectrical feedback via stretch-activated ion channels, establishing connexin-based gap junctions with surrounding myocytes and altering the orientation of cardiac lamellae. Thus, reentry is the most common (89%) mechanism for sustained Ventricular Tachycardia (VT) in DCM. Other mechanisms include increased automaticity and afterdepolarizations caused by abnormalities in ventricular myocytes’ resting potential (stretch-induced reduction in SERCA activity and backwards NCX function ZIPES) and assisted by the increased levels of circulation catecholamines [23,24]. 

## 5. How to Follow up the Progression of Diabetic Cardiomyopathy

Ejection fraction is used to assess LV function, and in the majority of situations, dictates the treatment of cardiovascular diseases [5]. However, LVEF as a measure of LV function has some important limitations. In particular, in DM patients, it loses its prognostic capacity, as there is an altered relationship between LVEF and death or HF in patients with diabetes. The risk for death or HF in a diabetic patient with an LVEF of 40% is equivalent to the risk in a non-diabetic patient with an LVEF of 25% [25]. 

Deformation imaging (strain and strain rate) using speckle-tracking echocardiography has been shown to be more sensitive than EF in detecting myocardial contractility [26]. A significant advantage over classical Doppler-based measurements of these parameters is the independence of speckle tracking from angle-related effects on measurements (whereas proper alignment is necessary in Doppler techniques). 

Following the introduction of deformation imaging, mainly including the study of tissue strain and strain rate, a more precise characterization of myocardial systolic and diastolic function has been made possible. More specifically, myocardial strain, longitudinal, circumferential and radial, has appeared to be relatively load-independent, given that they compensate for the initial condition of the myocardium [27]. 

The capability for real time three-dimensional imaging has allowed the inclusion of transplanar segment motion, occurring because of myofibrillar orientation that would have otherwise not been taken into account, simultaneously reducing examination time. Three-dimensional speckle-tracking-based deformation measurements have been shown to assess LV volume, function, dysynchrony and rotation, even when only subclinical derangements exist and can give more sensitive information of the effects of fibrosis than the estimation of EF. Thus, it has important prognostic value in the case of HFpEF [28]. However, a wide range of correlation has recently been noted between three-dimensional real-time speckle tracking voxel and tagged cardiac MRI (gold standard) circumferential strain measurements, emphasizing the need for further improvement [29]. The most important limitation in applying the above measurements is the requirement for regular cardiac cycles. 

Following combination of the aforementioned advances, echocardiography has been shown to carry significant prognostic value in DCM patients, regarding not only myocardial function but also arrhythmogenesis and sudden cardiac death [30]. Although segmental strain curves are synchronized in normal myocardium, in DCM, alterations in the curves’ form (signifying abnormal stretching in systole and post-systolic shortening in diastole), peak systolic values (result of reduced contractility) and dispersion of their peak values (highlighting the, often subclinical, dysynchrony) are evident [31].

The diagnosis of EAT expansion is warranted for the identification of EAT-related HFpEF phenotypes. Transthoracic echocardiography can assess EAT thickness, measured as the echo-lucent area between the epicardial surface and parietal pericardium, although it cannot be used to estimate EAT volume, having also relatively poor inter-observer and intra-observer variability among other limitations. The gold standard method for the assessment of EAT volume is cardiac magnetic resonance (CMR). In line with this, the European society of cardiology consensus recommends the use of a stepwise score-based algorithm to diagnose HFpEF [32].

## 6. The Role of N-Terminal-Pro Hormone BNP (NT-proBNP)

NPs, particularly brain natriuretic peptide (BNP) and atrial natriuretic peptide (ANP), are hormones secreted from cardiac chambers whenever an increased strain is imposed on them. Their main function is to promote natriuresis in the kidneys through direct inhibition of Na^+^ reabsorption, stimulation of their guanylyl cyclase receptor and inhibition of the renin–angiotensin–aldosterone axis and vasodilation induction through the latter mechanism. They have been used by clinicians in order to differentiate between dyspnea of cardiac and non-cardiac origin and may potentially assist in prognosis in the setting of heart failure, myocardial infarction and pulmonary embolism [33]. As in diabetic cardiomyopathy, there is an increased strain imposed on the left ventricle, and the use of natriuretic peptides (NPs) can be correlated with increased left ventricular diastolic pressures and LV hypertrophy. Thus, their use may act as prognosticators for the clinical course of patients. 

The use of the NPs has been proven in the initial diagnosis of patients presenting with shortness of breath and in the clinical management of patients with HF [34]. Furthermore, the long-term prognostic role of natriuretic peptides has been studied in subjects without clinical overt HF but with risk factors for HF development (ACC stage A) [34]. In a meta-analysis of primary data from 40 studies including 95,617 individuals without a history of CV disease, NT-proBNP strongly predicted first-onset HF and offered augmented risk prediction for coronary heart disease (CHD) and stroke [34]. The incremental predictive ability of NT-proBNP for CHD and stroke was greater than HDL cholesterol or CRP, and NT-proBNP could serve as a multipurpose biomarker in new approaches that integrate HF into CVD primary prevention. Additionally, in 16,492 patients with T2DM and a history of or at risk of CV events, a stepwise increased risk of hospitalization for heart failure was related with higher quartiles of baseline NT-proBNP values. There was a significantly increased risk of hospitalization for heart failure with the use of an established dichotomous cut-off point of 125 pg/mL (for age < 75 years) [34], whereas an absolute increase in NT-proBNP by 400 pg/mL was associated with a significantly elevated risk of CV events. In addition, dynamic changes in NT-proBNP over 6 months follow-up have shown a clear discrimination of risk and may be most pragmatic for clinical practice in high and low categories of NT-proBNP concentrations [34]. 

However, the mechanisms involved in the concept of DM-associated HF and the risk factors implicated in the prognosis of patients with DM are unknown. Assessing the risk in patients with DM is valuable not only for the identification of a high-risk subgroup but equally important also to define low-risk subpopulations. NP measurement by general practitioners (GP) and diabetologists in high-risk populations such as those with hypertension or T2DM aids in identifying patients with elevated ventricular diastolic pressures in order to strengthen the initiation of preventive measures, including medicine up-titration of renin–angiotensin system antagonists or the introduction of novel therapies such as sodium/glucose cotransporter 2 inhibitors (SGLT2i), and therefore, in preventing or slowing the development of HF [35,36,37].

The development of a standardized strategy to screen and intervene in patients at risk of developing HF is challenging, mainly due to the different definitions of HF risk, the heterogeneity of prevalence in different populations, the variable duration of subclinical course and the variable interventions for risk factor modification or treatment. Nowadays, it has become more obvious that the diagnosis of HF cannot be based only on clinical signs and symptoms, and markers reflecting hemodynamic condition, as elevated NP levels can determine HF diagnosis especially concerning heart failure with mid-range ejection fraction (HFmEF) and HfpEF [19,38]. Further studies are needed to determine the cost-effectiveness of such a screening and its impact on quality of life (QoL) and mortality.

At the moment, the focus should be on T2DM since it is perceived as a major risk factor for CVD and patients might benefit from an optimized treatment with renin–angiotensin system antagonists and β-blockers regarding cardiac events or the optimization/introduction of novel therapies. PONTIAC evaluated the primary preventative effect of optimal neurohormonal therapy in patients with DM without coronary artery disease, selected according to their higher values of NT-proBNP. The study showed that the primary end-point, which was hospitalizations or death, from heart failure was significant reduced in the group of optimal treatment in the two years of follow-up. Those findings need to be validated by more robust studies where key opinion leaders, payers and regulatory bodies may play a significant role [39].

**Figure 1:**
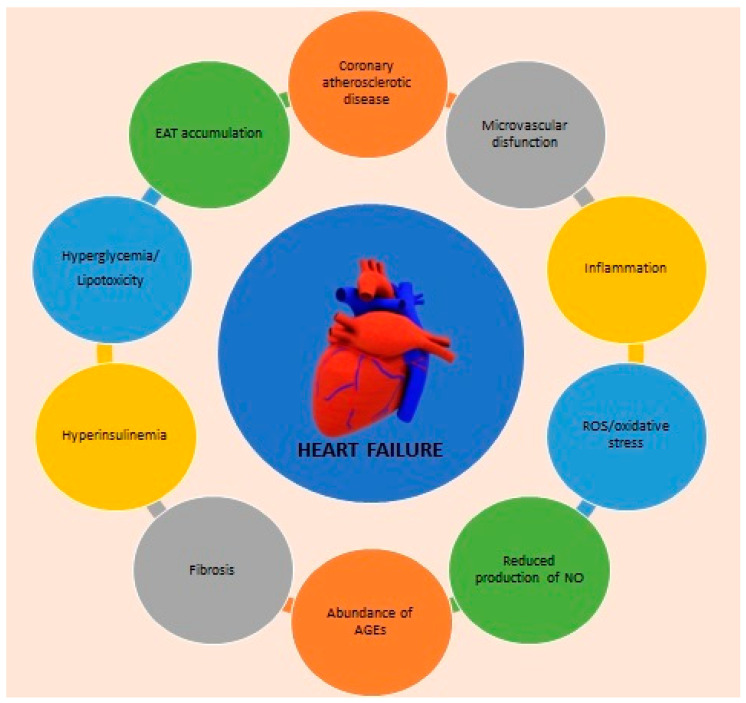
Multifactorial pathogenesis of heart failure in patients with T2DM. AGEs: Advanced glycation end products, EAT: epicardial adipose tissue, NO: nitric oxide, ROS: reactive oxygen species.

Both NT-proBNP and BNP have shown a predictive value for both short- and intermediate-term CV events in individuals with DM. Indeed, the SAVOR-TIMI 53 trial reported that diabetic individuals without known CVD but with elevated NT-proBNP levels had a 3-fold increased risk of HF development compared to counterparts with known CVD and normal NT-proBNP levels. Similarly, the ADVANCE and other trials demonstrated that NT-proBNP strongly predicts HF risk, the overall excess mortality and CV mortality in people with T2DM [40,41,42]. Similarly with the PONTIAC study, the STOP-HF trial supports the finding that BNPs are a reliable screening tool in an at-risk population identifying subjects who appear with HF or LV dysfunction. NT-proBNP levels also correlated significantly with the functional NYHA classes of HF [43]. However, despite their importance as prognostic tests, natriuretic peptides are still not used regularly in ambulatory care settings [38,44,45,46] Figure 1.

## 7. Lifestyle Interventions

Lifestyle interventions are a crucial step for the prevention and management of DM according to the American and European Guidelines [1]. Adaptation of an overall healthy lifestyle by diabetic patients has been related with a reduction in CVD mortality and morbidity, including hospitalizations due to HF, as shown in a recent study of 3804 diabetics of the ARIC population followed up for 26 years [47].

### 7.1. Weight Management

Data from cohort studies and a recent meta-analysis by Galaviz et al. including 17,272 patients [48] have provided evidence that a lifestyle modification targeting weight loss can prove beneficial in the prevention and management of T2DM. A modest weight loss of 5–10% has been associated with a significant reduction in HbA1c in diabetics and delayed onset of T2DM in prediabetics. Furthermore, it was related with an improvement in CVD risk factors, including blood pressure, thus implying a favorable impact in terms of the cardiovascular prognosis of these patients [1,49].

### 7.2. Physical Activity

Current guidelines recommend a moderate-to-vigorous physical activity of at least 150 min/week for the prevention and treatment of DM. A combination of aerobic and resistance training is the preferred regime as studies have demonstrated an additive benefit in terms of glycemic control, lipid profile and BP values. Furthermore, increased physical activity seems to delay the evolution of prediabetic conditions, such as IGT to T2DM, and improves CVD outcomes [1].

According to recent research data, exercise training in diabetic patients has been associated with an improvement in echocardiographic parameters assessing systolic and diastolic function and a further improvement in cardiorespiratory fitness as assessed via the cardiopulmonary exercise test (CPET) [50]. Interestingly, the beneficial impact of physical activity on myocardial function was even noticed in adolescents with T1DM [51]. These observations may imply a favorable effect of physical activity in the prevention and management of diabetic cardiomyopathy.

### 7.3. Diet

Nutritional guidance is an integral part of the management of every chronic condition. This is even more evident in the setting of a cardiometabolic disease such as DM. Many dietary patterns have been tried and suggested in diabetic populations, including low carbohydrate diets (LC), high protein diets (HP) and popular moderate macronutrient ones such as DASH and the Mediterranean diet. In diabetic patients, special dietary recommendations have been proposed according to individual caloric requirements, personal food preferences, medications administered, obesity status and the presence of other comorbidities. The intake of saturated fats should be reduced, while trans fat intake should be eliminated. A decrease in energy density is also recommended (<125 kcal per 100 g of food), while food should be enriched with whole grains, vegetables, nuts, poultry, legumes and vegetable oils [52]. Moreover, legumes, have a rich content in soluble and insoluble dietary fiber, reducing the rate of the absorption of glucose, are also excellent sources of peptides and phytochemicals with reported antidiabetic properties and can modulate the release of gastric hormones. Dietary habits play an important role in the composition of the gut microbiota, determining the microbial metabolites. Adherence to the Mediterranean diet were found to have beneficial microbiome-related metabolomic profiles. This is of extreme importance as specific metabolites have shown changes in their circulating levels prior to the onset of diabetes. Thus, the human gut microbiome profile can be modified by the action of dietary phytochemicals, implying probable bidirectional interactions [7,52,53,54,55,56,57].

Regarding LC diets, studies have provided controversial results [58]; however, there is evidence supporting their role in achieving better glycemic control and reduced insulin resistance. These effects contribute to a more favorable metabolic environment that could prevent the development of diabetic cardiomyopathy [59]. Usually, LC diets also imply a high protein intake. Although data are scarce, diets with high protein consumption have been associated with a significant improvement in major cardiometabolic parameters in diabetic patients with HF [60].

On the other hand, studies with moderate macronutrient diets in populations with or without DM have exhibited positive results in terms of cardiometabolic risk reduction. In a recent meta-analysis by Siervo et al., the DASH diet was related to a significant reduction in BP, total cholesterol and LDL, but it did not impact blood glucose and triglycerides [61]. Interestingly, Ge et al., in a meta-analysis including 21,942 participants, compared the cardiometabolic impact of 14 popular named dietary patterns on cardiovascular risk reduction and found that LC-HP diets, low fat diets and moderate macronutrient DASH and Mediterranean diets were associated with a significant reduction in weight and BP at 6 months compared to common diet plans. However, these beneficial effects seemed to fade away after 12 months, except for the sustained LDL reduction provided by the Mediterranean diet [62].

The Mediterranean diet has been widely studied for its cardiometabolic properties and its beneficial effects on general health. It is associated with an improvement in blood pressure control, lipid profile, glucose metabolism and arrhythmias. Therefore, the Mediterranean diet is suggested to play a significant role in reducing risk factors for heart disease, such as obesity, dyslipidemia, hypertension and diabetes [6,63]. For DM, in particular, the adoption of the Mediterranean diet has been linked to a reduction in fasting and postprandial glucose levels [58,59]. At the same time, it has been found that diabetic patients who follow the standard Mediterranean diet show lower levels of hemoglobin A1c (HbA1c) and improved insulin resistance [64,65]. 

Several research data and meta-analyses indicate that the various benefits of following a Mediterranean-style diet come from the synergistic effect of various nutrients of the diet and not from a single ingredient. The diet consists mainly of olive oil, fish, fruits, vegetables, legumes, and high-fiber unprocessed cereals. Thus, it is rich in monounsaturated and polyunsaturated fatty acids, fiber and antioxidants, while the intake of saturated fat is limited, and the amount of carbohydrate intake comes mainly from complex carbohydrates. All the above nutritional factors seem to contribute to a better control of glucose levels in patients with diabetes [66,67,68,69,70,71,72].

### 7.4. Smoking

**Figure 2:**
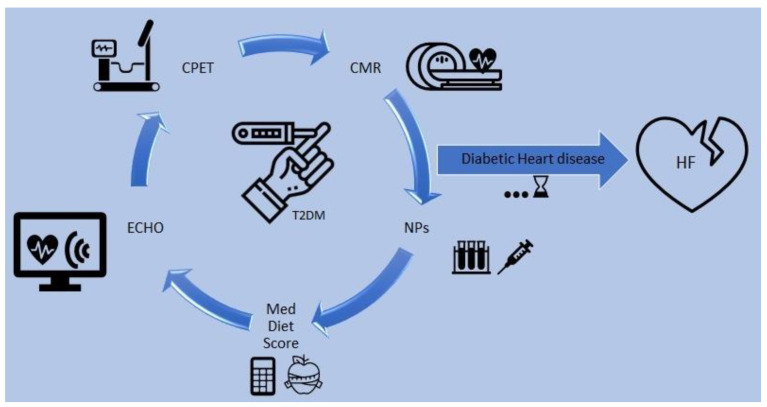
Prognostication of heart failure in patients with T2DM. CMR: cardiac magnetic resonance, CPET: cardiopulmonary exercise test, ECHO: echocardiogram, HF: heart failure, Med: Mediterranean, NPs: natriuretic peptides, T2DM: type 2 diabetes mellitus. The diagram presents a proposed evaluation of T2DM patients for risk stratification of heart failure, involving cardiac imaging, functional capacity, the Mediterranean diet score and biomarkers.

Smoking in the setting of DM has been associated with significantly worse outcomes regarding cardiovascular mortality and morbidity, including CAD, stroke, peripheral vascular disease and HF. Considering that smoking is another standalone classic risk factor for CVD, its cessation is imperative in this high-risk population [73]. Regarding electronic cigarettes, data are scarce and raise significant concerns regarding their cardiovascular impact in people with or without diabetes mellitus. Taking into account the heterogeneity of the included substances and the emerging phenomenon of dual-smokers (smoking both electronic and classic cigarettes), vaping cannot be safely suggested as an alternative or means of quitting smoking. Consulting a specialized smoking cessation clinic is warranted [74] Figure 2.

## 8. The Role of Medical Therapy—What Is an Effective Treatment?

There is a debate about the effectiveness of intensive glycemic control of DM patients in the primary prevention of HF. In a recent meta-analysis, intensive therapy for glycemic control did not provide an important impact on the risk of hospitalization due to HF [75]. 

Pioglitazone, a thiazolidinedione, increases the risk of developing heart failure, thus it has not gained any indication for use in HF patients, although it does not reduce cardiac function. Pioglitazone exhibits its main mechanism by promoting Na + reabsorption and fluid retention by activating sodium transporters in the proximal tubule and epithelial sodium channels in the collecting duct via peroxisome proliferator-activated receptor γ (PPARγ). Thus, its use in combination with a mineralocorticoid receptor antagonist or thiazide diuretics, can prevent fluid retention [76].

DPP4 inhibitors increase sympathetic nerve activity by increasing their bioactive proteins, which may be involved in the onset of heart failure. The SAVOR-TIMI 53 study, showed an increased risk of developing heart failure in the saxagliptin group [35]. Metformin is the most used medication in DM patients. Its main mechanism of action is by the activation of AMP-activated protein kinase (AMPK), which regulates energy metabolism in various tissues including the heart, liver and muscles, independently of blood glucose levels improvement.

Recently, it has been acknowledged that patients with HF share with DM patients’ similar pathophysiological characteristics, such as endothelium dysfunction, insulin resistance mitochondrial dysfunction, neurohormonal activation and inflammation. Thus, medications that were first used for the treatment of DM, gained recognition as medications for HF. In the EMPA-REG OUTCOME trial, empagliflozin, an SGLT2 inhibitor, was associated with a significant reduction in heart failure hospitalization and death in patients with T2DM with a history of CVD. Similarly, in the CANVAS trial, canagliflozin, another SGLT2 inhibitor, revealed a reduction in HF hospitalization in patients with T2DM at a high cardiovascular risk. Given these findings, SGLT2 inhibitors seem to be effective in preventing HF in patients with T2DM who have a history of CVD. There are two major mechanisms by which SGLT2 inhibitors prevent and improve the clinical status of HF, including metabolic and hemodynamic mechanisms. Metabolic mechanisms involve a hypoglycemic effect, protection from lipotoxicity, weight loss, increase in ketones in blood, decrease in insulin levels and improvement in insulin resistance. Hemodynamic mechanisms include a diuretic effect and decrease in blood pressure levels. An additional mechanism is the down-regulation in sympathetic activity [77,78]. Additionally, Empagliflozin seems to slow the progression of kidney disease and reduce the risk of renal events compared with a placebo. 

Regarding other treatment, all patients with DM, in addition to metabolic control, should receive treatment with an angiotensin-converting enzyme (ACE) inhibitor (unless contraindicated) regardless of the level of left ventricular dysfunction.

The HOPE (Heart Outcomes Prevention valuation) study, in a population of 9297 high-risk patients (3577 diabetics), illustrated significant benefits for both cardiovascular morbidity and mortality with ramipril, but this benefit was even more impressive in DM patients.

The only large ACE-inhibitor trial in HFrEF to provide detailed information on patients with T2DM was the ATLAS, which compared low-dose (2.5–5.0 mg daily) to high-dose (32.5–35.0 mg daily) lisinopril. The occurrence of adverse effects with high-dose lisinopril was similar in those with and without T2DM with respect to hypotension/dizziness (35% vs. 32%), renal dysfunction/hyperkalemia (29% vs. 22%) and cough (12% vs. 10%). The possible preventive effect of losartan in diabetic patients with type 2 diabetes was evaluated in the subset analysis of two large, randomized trials: RENAAL for renal protection and LIFE for hypertension with left ventricular hypertrophy. 

Compared to a placebo, losartan significantly reduced the incidence of the first hospitalization for HF: 39 versus 54% in RENAAL (adjusted HR 0.69), and 11 versus 19 percent in LIFE (adjusted HR 0.50).

There is little information about the tolerability of angiotensin receptor blockers (ARBs) in T2DM. In the overall CHARM program, patients with T2DM had double-fold the risk of developing hyperkalemia on candesartan compared to those without T2DM.

In the CHARM trial, a significant reduction in CV death, HF hospitalization and all-cause mortality was achieved with candesartan in patients with HF and HFrEF, irrespective of the presence of T2DM [79,80,81]. 

## 9. Conclusions

The mechanisms involved in the concept of DM-associated HF as well as prognosticator factors are multifactorial. Assessing the risk in patients with DM is valuable not only for the identification of a high-risk subgroup but also to define low-risk subpopulations. Nowadays, DM and HF have been recognized as sharing similar metabolic pathways; thus, the clinical manifestation of HF can be independent of LVEF classification. Consequently, approaching HF should be through structural, hemodynamic and functional evaluation of each patient.

A predictive model that includes imaging data, functional capacity index, lifestyle factors, such as a score for a health diet, and biological markers could give information on the risk stratification of DM patients. Identifying high-risk patients among those with DM that are prone to the occurrence of HF, can establish the initiation of more intense preventive measures, including medicine up-titration of renin–angiotensin system antagonists or the introduction of novel therapies of HF for patients with preserved ejection fraction. The promotion of a healthy lifestyle including the cardioprotective Mediterranean diet, regular exercise and lack of smoking, is always encouraged in order to slow the development of HF.

## Abbreviation

Adenosine triphosphate	ATP
Advanced glycation end products	AGEs
Atrial natriuretic peptide	ANP
Brain natriuretic peptide	BNP
Cardiac magnetic resonance	CMR
Cardiovascular disease	CVD
Cardiovascular health	CV
Coronary artery disease	CAD
Coronary heart disease	CHD
Cyclic guanosine monophosphate	cGMP
Diabetes mellitus	DM
Dilated cardiomyopathy	DCM
Ejection fraction	EF
Epicardial adipose tissue	EAT
General practitioner	GP
HbA1c: hemoglobin	A1c
Heart failure	HF
Heart failure with mid-range ejection fraction	HFmEF
Heart failure with preserved ejection fraction	HFpEF
Interleukin	IL
Left ventricular	LV
Left ventricular ejection fraction	LVEF
Magnetic resonance imaging	MRI
Monocyte chemoattractant protein-1	MCP-1
Natriuretic peptides	NP
Nitric oxide	NO
N-terminal-pro hormone BNP	NT-proBNP
Plasminogen activator inhibitor-1	PAI-1
Protein kinase G	PKG
Quality of life	QoL
Reactive oxygen species	ROS
Sodium/glucose cotransporter 2 inhibitors	SGLT2i
Tumor necrosis factor alpha	TNF-α
Type 2 diabetes mellitus	T2DM
Ventricular Tachycardia	VT
